# On stimulus persistence and human behavior: the stimulus persistence unification theory

**DOI:** 10.1080/19420889.2022.2141954

**Published:** 2022-11-07

**Authors:** Tobore Onojighofia Tobore

**Affiliations:** Independent Scholar

**Keywords:** Stimulus persistence, human behavior, conflict, Russia-Ukraine war, international relations, politics, North Korea, international sanctions, side effects, chronic diseases, chronic pain, opioid crisis, COVID-19, COVID variants, pandemic, dangerous stimuli, unpleasant stimuli, escalation, treatment escalation, nuclear war, nuclear escalation, UN security council

## Abstract

A person trapped in a building engulfed in a raging fire, a person dealing with severe chronic disease, people dealing with a virus pandemic, and people fighting in a protracted war may appear dissimilar but are fundamentally in a similar situation and their behaviors follow a predictable and similar pattern. In this paper, the behaviors of rational people dealing with a significant persistent unpleasant, or dangerous stimulus that is inescapable are elucidated. The unique modulatory effects of stimulus persistence on human behavior as well as the role of means and interest are discussed.

## Introduction

The ability to respond to a stimulus, defined as any internal or external agent, event, or situation that provokes a response from an organism, is a distinguishing characteristic of living things [1]. Response to stimulus is crucial to an organism’s survival and reproduction [1] and in response to stimuli, organisms adapt their physiology and behavior [2]. Indeed, stimuli play an important role in animal behavior including habits [3] learning, and decision-making [2].

Different types of environmental stimuli modulate animal behavior including pressure, temperature, salinity, light/darkness, displays, calls, movements, pheromones, colors, etc. [2]. Response to stimuli that are unpleasant or threatening usually evokes a psychophysiological reaction promoting the organism’s readiness for active defense and enhanced conflict monitoring [4,5]. Response to an unpleasant or threatening stimulus is typically to ignore it when it is insignificant and familiar and to escape or withdraw when it is significant and/or novel. However, when an unpleasant or threatening stimulus is significant and/or novel and inescapable, the response is to try to survive or ensure well-being by taking steps to neutralize or render it impotent. When efforts to counteract this stimulus are unsuccessful, it is considered persistent and its continuing presence has a significant effect on human behavior.

The objective of this paper is to discuss the effects of the persistence of a significant and/or novel inescapable unpleasant or dangerous stimulus on human behavior.

## Relationship between stimulus persistence and interests and means

Two important factors play a crucial role in the relationship between a threatening or unpleasant persistent stimulus and human behavior: interest and means. Interest entails a person’s desire or proclivity to counteract a stimulus. Typically, interest to counteract a novel and/or significant unpleasant or threatening stimulus is high. However, if the stimulus becomes persistent because efforts to neutralize it are unsuccessful, this modulates interest and behavior. Interest to counteract an inescapable persistent dangerous or unpleasant stimulus is influenced by the perceived severity and strength of the stimulus before, during, and after the employment of the neutralizing intervention. Interest is also modulated by different factors including, negative consequences, knowledge and education, trust, group cohesion or oneness of purpose, beliefs [6,7], benefit/harm relative to energy expended toward counteracting the persistent stimulus, etc. Interest to counteract a significant and/or novel threatening or unpleasant inescapable stimulus may be low but it is never zero.

As Table 1 shows, interest can be categorized into four classes. Class 4 is interest at its highest. At this point, interest to counteract the stimulus is existential and very resistant to modulation.

**Table 1.** Classes of interest.

Levels of interest	Definition
Low (Class 1)	Weak interest to continue efforts to counteract a dangerous or unpleasant stimulus.
High (Class 2)	Adequate interest to counteract a dangerous or unpleasant stimulus
High (Class 3)	Strong interest to counteract a dangerous or unpleasant stimulus
High (Class 4)	Monumental interest to counteract the stimulus The threat posed by the stimulus is perceived as “existential”

Means, on the other hand, entails the resources available including social support, finances, access, manpower, capability, and ability to help counteract or neutralize the stimulus. Means impact interest and the interplay between them triggers different behavioral responses to a persistent stimulus.

**Figure 1.**
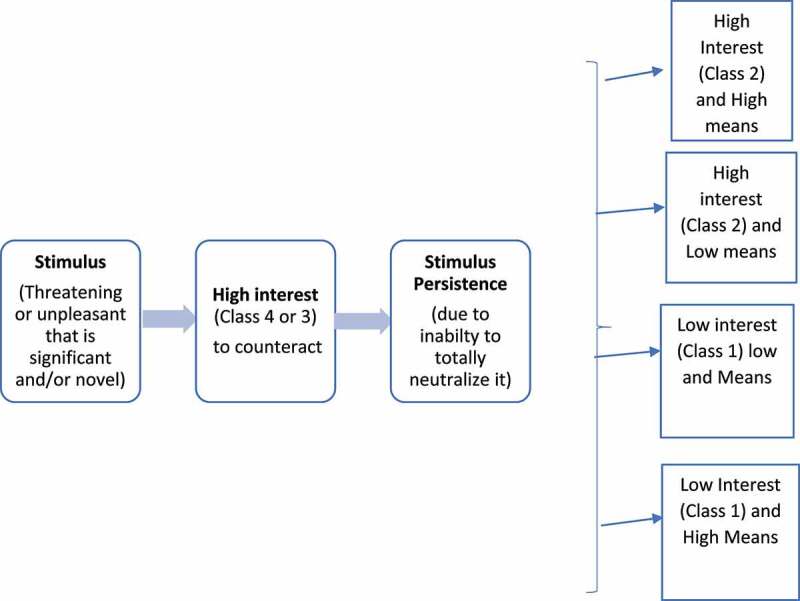
Modulatory effect of stimulus persistence on interest and means.

As Figure 1 shows, initial interest to counteract a significant and/or novel inescapable stimulus is typically Class 3 or 4 regardless of means. If the stimulus becomes persistent, interest is modulated by many factors including means, individual perception of the threat or unpleasantness of the stimulus, burden imposed by the stimulus, the effectiveness of the neutralizing intervention and the benefit/harm of maintaining the neutralizing intervention. There are several potential outcomes of the persistence of an unpleasant or dangerous stimulus; high interest and high means, high interest and low means, low interest and low means, and low interest and high means.

**Figure 2.**
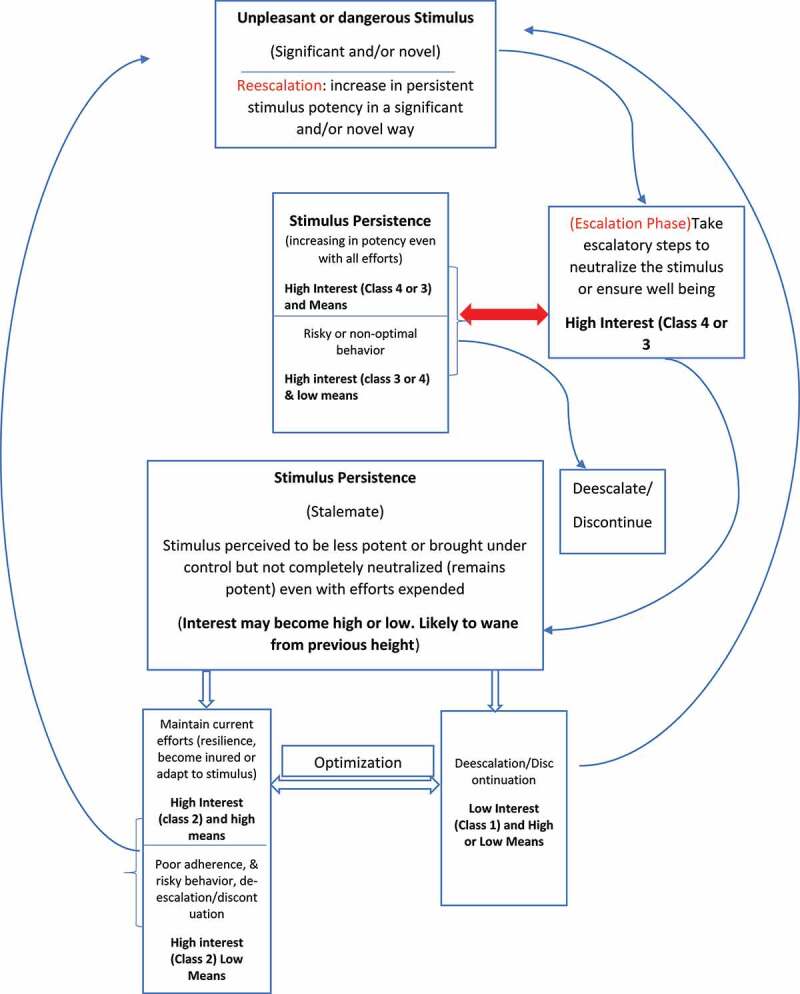
Persistent stimulus impact on interest, means, and behavior.

Figure 2 provides insight into how stimulus persistence modulates behavior and how interest is modulated by different factors.

### Escalation phase (high interest, Class 4 or 3)

Escalation can be defined as a significant and rapid increase in efforts and measures to counteract or neutralize a threatening or unpleasant stimulus or a significant change in the potency or severity of the stimulus. If faced with a novel and/or significant dangerous or unpleasant stimulus that is inescapable, the objective of the stimulus receiver (the person dealing with the stimulus) would likely be to escalate to neutralize the stimulus. As an example, at the beginning (March 2020) of the COVID-19 pandemic [8], there was a general effort to counteract the virus by taking preventive measures and evidence suggests that all people were alike in their likelihood of taking part in preventive personal behaviors [9]. Similarly, uncontrolled chronic disease symptoms result in treatment escalation in efforts to control it, and this is associated with increased health-care costs [10–12]. Also, significant worsening in chronic patient disease severity typically leads to treatment escalation to counteract the worsening [13,14]. Correspondingly, in a conflict, many civilians counteract the dangers of bombs and missiles by hiding in the subway or their basements [15,16].

### Stalemate phase (high interest, Class 2)

Over time, if a persistent unpleasant or threatening stimulus is perceived to be less potent, brought under control, or stabilized, it enters a stalemate phase. If the stimulus is stabilized or perceived to be reduced from its initial potency, this may indicate that current efforts to counteract it are helpful. In this situation, interest to continue to counteract the stimulus is likely to wane from its previous high (3 or 4) before the attenuating intervention. Indeed, during the COVID-19 pandemic, after the introduction of preventive measures, evidence suggests that over time there was an increased likelihood of risky behavior across groups [9,17]. Multiple lines of evidence support the notion of fatigue or decreased motivation to follow pandemic-instituted social distancing measures and health-protective behaviors over time [18–20]. Similarly, patient treatment adherence and persistence in many chronic diseases tend to decrease significantly over time if their symptoms are brought under control. Indeed, forgetfulness and patient good self-perceived health which are typical when disease symptoms are under control are known to play in role in reducing medication adherence and persistence [21].

If a persistent stimulus is perceived to be less potent, under control or manageable, resilience, habituation and adaptation are likely to follow. Indeed, after several years of people dealing with COVID-19, the availability of vaccines and preventive measures to counteract it has increased the need to live with it as part of daily life [22], indicating that people have come inured or resilient to its presence. Similarly, evidence suggests that over time many patients suffering from chronic disease/condition develop coping/adaptive mechanisms and resilience, and those who develop these mechanisms display quicker recovery from the negative effects of their condition [23–27]. These adaptive mechanisms developed to counteract the persistent unpleasant symptoms of the disease include prayer/meditation, optimistic outlook, social support, consistent exercise, being engaged in the community, expressing a greater belief that their lives have meaning, and accepting their condition and its consequences [23,24,28–31]. In contrast, lower acceptance of illness is associated with poorer health conditions [32].

In the stalemate phase, interest to maintain efforts to counteract the persistent stimulus is modulated by multiple factors including the individual perception of the potency of the persistent stimulus, socioeconomic status (SES), cost or stringency of the intervention, education/literacy, perceived benefit/harm of the attenuating intervention, etc. Research indicates that patient treatment adherence and persistence in many chronic diseases tend to decrease significantly over time for multiple reasons including forgetfulness, age, side effects, comorbidities, good self-perceived health, mood, and patient knowledge about medication regimen [21,33–36]. Similarly, people who saw the threat from COVID-19 as significant were more likely to maintain compliance with preventive measures [37,38]. Indeed, being female [37], having higher SES, having a higher perceived risk for infection and dying, having a more left-leaning political orientation, and having more COVID-19 cases in one’s state of residence positively influenced the likelihood of following or adopting pandemic mitigating behaviors [9].

### Persistent stimulus increasing in potency (high interest, Class 3 or 4)

If a persistent stimulus is perceived to be increasing in potency, there is a tendency for interest to counteract it to remain very high (3 or 4) and this increases the likelihood of continued escalation of efforts to counteract or neutralize the stimulus regardless of whether means is low or high. Indeed, escalation of efforts to contain or suppress a rapidly spreading wildfire in the city area will continue as long as necessary to save lives and properties. The 2017 wildfires in California saw one of the worse destruction of properties and lives and the state mobilized a large number of firefighters and enormous resources to suppress the fire [39,40]. Until the fire was suppressed or brought under control, there was continued escalation. Similarly, uncontrolled chronic disease symptoms result in continuous treatment escalation in efforts to control it, and this is associated with increased health-care costs [10–12]. Research indicates that high levels of persistent pain despite the use of analgesics translate into a lower adaptation to illness leading to a decrease in the acceptance of illness [32]. This indicates that resilience and adaptation are less likely for patients with uncontrolled pain or those suffering from pain that is worsening in potency despite all efforts to attenuate it and their lower adaptation, reduced acceptance of illness, and lower life satisfaction makes them more likely to continue to escalate efforts to reduce or counteract their pain.

### De-escalation or discontinuation (low interest, Class 1)

When a persistent stimulus is perceived to have reduced in potency, been stabilized, or brought under control, interest to continue the stimulus neutralizing intervention could become low [1] regardless of whether means is high or low and this could result in discontinuation or de-escalation of the stimulus neutralizing intervention. Indeed, long-term treatment may be perceived by chronic patients as a loss and burden and this can negatively impact treatment adherence [41] regardless of SES. Evidence suggests that many chronic disease patients develop depression which could hamper their adherence to treatment [42–44]. Forgetfulness and patient good self-perceived health which are typical when disease symptoms are under control are known to play in role in reducing medication adherence and persistence [21]. Correspondingly, the nearly 20-y war of the US in Afghanistan was discontinued in 2021 [45] even though the country had the means to continue the war largely because of internal reevaluation of the country’s interest after significant human and financial resources have been expended and the perception of reduced threat level from Afghanistan.

Also, if a stimulus is worsening in potency and it is impossible to bring under control or neutralize it, this could lead to a reduction in interest to Class 1 and discontinuation or de-escalation of the attenuating intervention. As an example, treatment for terminally ill patients reaches a point where nothing more can be done for the patient but palliative care [46,47]. At this point, treatment is de-escalated as it may contribute to the patient’s suffering, and end-of-life palliative care is recommended. Similarly, physician-assisted suicide or medical assistance in dying is typically requested by patients with irremediable and debilitating medical conditions, who have lost hope and for which further treatment will not be significantly helpful to improve their grievous symptoms or quality of life [48–50]. Correspondingly, the Soviet Union’s invasion of Afghanistan in 1979 was met with stiff prolonged resistance that they were unable to overcome. Change in leadership with the emergence of Mikhail Gorbachev brought about a significant change in the country’s perceived interest and coupled with the unsustainable cost of the war, the significant loss of troops and equipment, prompted the Soviet Afghanistan withdrawal [51,52]. The same situation applies to the US–Vietnam war where the US spent over $200 billion and lost about 58,000 people [53] without neutralizing the communist North Vietnamese army.

Moreover, in the stalemate phase, interest in a stimulus neutralizing intervention could move between Class 2 and Class 1 due to intervention optimization. As an example, costs, side or adverse effects of medication, and any positive change in disease risk assessment could spur treatment discontinuation, dose reduction, or the use of alternative medications without any treatment escalation [54,55].

### Re-escalation

In the stalemate phase, a significant change in the potency and/or novelty of the persistent stimulus increases the likelihood of escalation of efforts to counteract it. As an example, although, there have been many variants of COVID-19 [56], only the ones that posed a significant and/or novel threat led to the implementation of escalatory preventive measures which were removed once the threat was considered manageable. Indeed, the emergence of the more potent delta variant of COVID-19 spurred global panic causing many countries to reinstate precautionary measures such as lockdowns, curfews, travel restrictions, and mask mandates that have previously been lifted [57]. Similarly, the emergence of the omicron variant, a novel, and significantly dangerous variation of the virus, spurred global panic and the reinstatement of precautionary measures [58,59].

This tendency to escalate efforts to neutralize a persistent stimulus when it increases its potency is similar to what is observed in the treatment of patients with chronic illnesses. Patients suffering from chronic diseases which typically have no cure have to contend with many grievous symptoms and treatment typically follows an individualized approach which is guided by the patient’s response to treatment, patient’s disease severity, clinical status, and preferences [60,61]. The initial regimen is to stabilize the patient’s condition and maintain it if the patient is doing well. Treatment escalation is typically recommended if there is clinical or disease worsening until the maximum that can be done for the patient is reached. Patient treatment adherence and medication persistence, treatment response, and clinical status are actively monitored and treatment is altered as necessary to achieve the best possible outcome for the patient [60]. Correspondingly, escalation in a conflict or war typically follows the same pattern. Indeed, the US military escalatory approach (increasing troop levels which was popularly known as the surge) was employed to arrest a worsening situation in both Iraq in 2007 and Afghanistan in 2009 [62–64].

### Stimulus persistence: risky and non-optimal behavior (high interest (Class 4, 3, and 2) and low means)

If interest is very high (4, 3, or 2) but means is low, this increases the tendency for risky or reckless behavior and non-optimal approach. When interest is extremely high (Class 4) and means are low is called “grave” point defined as the most dangerous point for risky or non-optimal behavior. A good example of a grave point situation is that of people caught in the raging fire at the world trade center towers. They had a Class 4 (existential) interest to counteract the fire but low means and many chose to jump from the World Trade Center towers rather than face death by asphyxiation or fire [65], a non-optimal choice. Correspondingly, a nation-state like North Korea that perceives existential threats to its existence has developed a nuclear program in response to these perceived threats. The international community and the United Nations have imposed heavy economic sanctions which have significantly reduced the means (access, finances, and resources) of the country. These sanctions have not changed the behavior of North Korea because interest remains extremely high (Class 4). The reduction in means has made the North Koreans develop methods to evade the sanctions and counteract the perceived existential threats by engaging in non-optimal or non-conventional behavior expected from a nation-state [66] including cyberattacks, brinksmanship, nuclear and missile tests [67,68], and crypto theft [69].

Another example of a grave point situation is that of patients addicted to opioids. The symptoms of opioid withdrawal following addiction are intolerable for many patients. Indeed, the desire to avoid withdrawal symptoms and feel normal are the most important factors that drive drug-seeking behavior [70]. The approach for some physicians who suspect a patient of opioid misuse or abuse is to discontinue opioid therapy and even terminate the relationship [71]. The problem is that the withdrawal symptoms and cravings which have been described as life-threatening [72], are overwhelmingly strong that the interest to feel normal and counteract the symptoms is extremely high (Class 4). Cut off from prescription opioids, many patients switch to heroin and illicitly produced fentanyl [73] to counteract these withdrawal symptoms and feel normal. Some opioid addicts take to theft [74,75] and some women turn to prostitution [76] to satisfy this powerful interest. Also, opioid addicts incarcerated face imposed withdrawal, and to alleviate their withdrawal symptoms some took medications prescribed for other patients or bought “spitback” medications [77].

If a persistent stimulus is perceived to be less potent or stabilized and interest is high (Class 2) but means is low, this increases the tendency of risky behavior and non-adherence or compliance. Indeed, if means is low because the proposed intervention is too tasking or expensive, adherence is likely to be reduced. Research indicates that low SES was found to be associated with more risky behaviors and difficulty practicing recommended COVID-19 pandemic mitigating behaviors [9] and high-cost COVID preventive measures (physical distancing) saw a decline in adherence [17]. Similarly, evidence suggests a strong relationship between treatment discontinuation over time and SES [78–81].

## Discussion

Interests and means are the cornerstones for understanding the influence of the persistence of a significantly threatening or unpleasant inescapable stimulus and their variations in efforts of counteracting the stimulus provide an insight into the dynamism of human behavior.

If an unpleasant or threatening stimulus is worsening in potency, there is an increased likelihood of poor adaptation and high interest to continue the escalation of efforts to neutralize it to ensure well-being. However, if a persistent unpleasant, or threatening stimulus is perceived to be less potent, or brought under control (stabilized), interest to counteract the stimulus is likely to reduce from its previous high before the attenuating intervention. Interest to maintain the attenuating intervention is modulated by different factors and may reduce over time. Interest follows this pattern in all persistent inescapable unpleasant or threatening stimuli (Figure 2). Indeed, the introduction of COVID-19 preventive measures caused an increase in risky behavior across all groups, and adherence to COVID preventive measures reduced over time because of reduced perceived risk, the cost/stringency of preventive measures, knowledge/literacy, and SES. Correspondingly, medication adherence reduces over time in chronic diseases due to the perceived benefit/harm of the medication, good self-perceived health, knowledge/literacy, SES, and side effects. Similarly, interest to maintain support for Ukraine by western allies in the Russian-Ukrainian conflict [82] may reduce over time due to western allies’ economic damage, inflation, high cost of living, and Ukrainian success or failure on the battlefield. When a persistent inescapable stimulus is perceived to be less potent or stabilized, escalation of efforts to neutralize the stimulus is usually when the threat or burden from the stimulus becomes significant and/or novel in its potency and form. If the stimulus change in potency is not perceived as significant, there is a low likelihood of escalation.

Also, interest to counteract any persistent inescapable unpleasant, or threatening stimulus follows the same pattern of escalation and de-escalation/discontinuation. Indeed, just as mutations of COVID-19 into more significantly dangerous strains led to an escalation of preventive measures to counteract it so is the treatment of chronic disease escalated when there are signs of significant clinical worsening and de-escalated/discontinued in disease remission or when symptoms severity is reduced/stabilized. Similarly, a protracted war between two evenly matched opposing forces will result in a stalemate and a significant escalation from one of the parties will elicit a similar response from the other. Furthermore, just as interest reduces for further treatment if a chronic disease reaches a point where nothing can be done for a patient so does interest to continue fighting reduce resulting in discontinuation if one of the warring parties in a conflict is overwhelmed by the stronger force. Just as side effects or perceived lack of benefit of medication in the treatment of a chronic disease results in low interest and discontinuation so is poor food or supplies and inferior weapons to face a superior enemy in a war can lead to personnel low interest to fight and desertion.

Risky and non-optimal behaviors are common when interest is high and means are low and the same pattern is observable in all situations involving a persistent inescapable unpleasant or dangerous stimulus. Of note is Class 4 interest which is extremely difficult to modulate. Risky behavior is highest at grave point; the point where a stimulus receiver has a Class 4 interest but low means. The unfortunate case of a patient in persistent pain with no relief from treatment who took the extraordinary step of murdering his physician [83] is a case in point of how extremely high interest to counteract a significant and inescapable persistent stimulus coupled with low means can push people to act in non-optimal ways. However, although extremely difficult to modulate, if means are significantly reduced, a Class 4 interest can be modulated in some cases. As an example, the North Korean regime which perceives an existential threat from the US, conducted a nuclear test in 2006 which was followed by economic sanctions from the UN security council. During this time, evidence suggests that trade between North Korea and its main economic partner remained largely unaffected [84]. Unsurprisingly, they conducted further tests in 2009, 2013, twice in 2016, and 2017. Importantly, in 2018, North Korea stopped its missile programs (long-range nuclear and ballistic) and indicated that it was willing to denuclearize. Research indicates that a decline in trade between North Korea and her economic benefactor, China, and improvements in the China-US relationship modulated North Korea’s behavior [85] during this time. Another example is the Portuguese colonial war (1961 and 1974) stopped because of the significant depletion of means (the war was consuming significant state resources) which spurred a change in internal leadership and revision of the country’s strategic interest [86]. Similarly, some opioid addicts incarcerated with no access to heroin were forced to detox from heroin [77].

The stimulus persistence theory can serve as a framework to predict behavior and could be used as an interventional framework. The ongoing war in Ukraine [87] is a case in point. The Russian plan to neutralize the Ukrainian army was met with significant resistance and this has prolonged the conflict [82]. Significant economic sanctions have been imposed on Russia [88] aimed at depleting her means (finances and resources) to finance and prosecute the war but this has not been successful in modulating her interest; a Class 4 interest that is very resistant to modulation. Russia’s means have not been depleted as the high energy prices have cushioned the effects of the economic sanctions and not all countries have supported the sanctions [88]. Ukraine is being supported with resources to continue fighting. Using the stimulus persistence theory, below are some possible outcomes of the conflict;

1. The sanctions will ultimately take effect significantly reducing Russian financial means. This may result in the use of non-optimal methods and weapons to achieve their objectives if interest remains extremely high.
2. Casualties in the war on one or both sides eventually impact means (manpower) and modulate interest increasing the likelihood of negotiation and de-escalation.
3. Change in leadership by either warring party could reduce interest from Class 4
4. The war falls into a stalemate and Ukrainian allies become inured to the fighting and this may reduce their interest from its height at the time of the invasion.
5. Interest of Ukrainian allies is reduced because of the economic damage/pain caused by the sanctions/war and a lack of Ukrainian success with the significant resources and energy expended.
6. Russia makes significant advances with the potential for total defeat and Ukrainian allies escalate because interest remains very high.
7. Ukraine makes significant advances with the potential for total defeat and Russia escalates because interest remains very high.
8. Russia makes significant and steady advances overwhelming the Ukrainians leading to negotiations and de-escalation.
9. Facing significant losses and potential defeat, Russia employs nuclear weapons or a new tactic that causes unprecedented, outrageous, and significant damage to Ukraine resulting in escalation from Ukraine and her partners

The economic sanctions will not stop the war as far as Russia continues to perceive Ukraine and its pro-western tilt as an existential threat. Indeed, reducing the means could potentially make things worse if Russia’s interest remains very high. Alternative weapons, tactics, and brutality may increase as a consequence of low means and extremely high interest. As far as Russian means are not significantly reduced, it will be difficult to change her interest level. The possibility of change in leadership on both sides is impossible to predict but this could potentially impact interests and objectives.

Also, chronic diseases are on the rise and are expected to rise further in the future [89]. Many of these diseases have no outright cure and the treatment goal is often to improve patient quality of life and relief symptoms. Patients with chronic conditions whose interest to attenuate their symptoms remains high or extremely high (Class 4 or 3) are more likely to be those who are not getting effective relief for their most burdensome symptoms, are unable to develop adaptive mechanisms, and accept their illness. Such patients are likely to suffer from low quality of life and low life satisfaction. The goal of treatment of patients with chronic disease or conditions must be to reduce interest to Class 2 by effectively relieving patients of their most severe symptoms, helping them adapt and accept their illness/condition, and employing resources including patient education, psychosocial support, psychological treatment, peer support, etc. Ways to help patients find meaning in life should also be explored because evidence suggests that the presence of meaning is negatively associated with negative affect and positively associated with life satisfaction, and happiness [90]. Patients whose interest remains in Class 3 or 4 (those with greater disease burden or unattenuated symptoms) with low means may engage in risky non-recommended or non-optimal behaviors to relieve their condition including non-evidence-based approaches (such as traditional and herbal medicine), suicide, and even violence. The government must do more to help patients with severe chronic conditions particularly those of low SES to have optimal access to healthcare and relevant health services as well as provide financial support. Clinicians must do all they can to attenuate patients suffering and reduce their interest to counteract the unpleasant persistent stimulus of their chronic condition from being too high (Class 4 or 3) or too low (Class 1).

Finally, it is important to note that the stimulus persistence theory works on the assumption that people (the stimulus instigator if it is a person and the stimulus receiver) are rational actors because the actions of irrational actors are unpredictable. Also, its scope is limited to when a person is dealing with a significant unpleasant or dangerous persistent stimulus that is inescapable. There are variations in the definition of stimulus persistence from a few seconds/minutes in a case of a person trapped in a building by a raging fire to years in the case of a protracted war. Furthermore, individual perception of the significance of a threat or unpleasantness of a stimulus varies and this may affect individual response. Learned helplessness which tends to develop with prolonged exposure to uncontrollable and persistent stressors could play a role in individual interest to counteract a persistent significant unpleasant or dangerous stimulus [91,92]. Human decision-making is complex and interest is modulated by different types of biases and fallacies that may not have been comprehensively discussed in this paper. Also, the theory applies to humans and may not be applicable in understanding behavior at the cellular or micro levels. Although the theory may be used to understand and predict the behavior of rational people dealing with a significant persistent aversive stimulus that is inescapable, the evidence presented in this paper includes the behaviors of countries in international relations, behaviors of people dealing with addiction, severe chronic diseases/conditions, wildfire, a virus pandemic, and a protracted conflict. There may be situations of rational people dealing with an inescapable significant persistent aversive stimulus that the theory has not taken into account and in which it may not be applicable. The most important implication of this theory is that it unifies dissimilar situations involving a persistent and inescapable stimulus improving understanding of behavior in these different situations. Another implication of the persistence theory is that interest can be created or elevated. However, over time, interest to continue to counteract or maintain the attenuating intervention is likely to wane due to various factors including reduced perception of the stimulus threat potency, lack of perceived benefit with efforts expended or required to be expended, literacy/education, cost or stringency of neutralizing intervention, etc. The most effective way to counteract this tendency and increase interest again will be to make the stimulus appear significantly more threatening or unpleasant especially in a novel way, or stimulate interest using different approaches including increased education, and trust-building measures such as engagement, improved messaging on the benefits of counteracting the stimulus and maintaining the attenuating intervention measures, reducing the cost/stringency of the attenuating intervention, especially for people of low SES, etc.

## Conclusions

The behaviors of rational people dealing with a significant persistent aversive stimulus that is inescapable are similar and predictable. A person managing a severe chronic disease is fundamentally in a protracted war with the disease and their behavioral patterns are similar to people in a state of war or in an area where there is a significant and dangerous wildfire or disease outbreak. Interest to counteract a significant persistent threatening or unpleasant stimulus is influenced by many factors including the individual perception of the threat or unpleasantness of the aversive stimulus, means to counteract it, the effectiveness of the neutralizing intervention well as the cost/benefit of maintaining the neutralizing intervention.

## The shock of the new member

A person who joins a group that has become inured or resilient to an unpleasant inescapable persistent stimulus is likely to be shocked at the indifference displayed by the group members toward the stimulus. However, over time being in the group, as far as means are low and the new member cannot galvanize the interest of the group to counteract the stimulus, they may gradually become inured to the stimulus. As an example, India is a country with a persistent problem of urban waste management driven by low interest and means. Indeed, this problem is believed to be due to inadequate policy and legislative tools as well as low financial resources, and capacities of local bodies [93]. Both the local government and the populace have become indifferent to the problem. A foreigner visiting this area of India will be shocked at the way people ignore the waste problem and go about their lives. If the foreigner has no choice but to live in this area for a long time, they may initially escalate efforts to counteract the unpleasant stimulus emanating from the waste by using a mask and other methods. But over time, if the stimulus is unvarying or not perceived to be worsening, and if the attenuating measures are burdensome, they may not maintain the attenuating measures. They are likely to adapt and become inured or indifferent to the conditions.

## References

[1] Agutter PS, Wheatley DN. Responding to the environment. About Life [Internet]. 2007;79–89. [cited 2022 Jun 3]. Available from: https://link.springer.com/chapter/10.1007/978-1-4020-5418-1_9

[2] Manière G, Coureaud G. Editorial: from stimulus to behavioral decision-making. Front Behav Neurosci. 2020 Jan 10;13:274. 31998089 10.3389/fnbeh.2019.00274 PMC6965054

[3] Thrailkill EA, Trask S, Vidal P, et al. Stimulus control of actions and habits: a role for reinforcer predictability and attention in the development of habitual behavior. J Exp Psychol Anim Learn Cogn [Internet]. 2018 Oct 1;44(4):370–384. [cited 2022 Jun 10]. Available from: https://pubmed.ncbi.nlm.nih.gov/30407063/30407063 10.1037/xan0000188 PMC6233324

[4] Buodo G, Sarlo M, Mento G, et al. Unpleasant stimuli differentially modulate inhibitory processes in an emotional Go/NoGo task: an event-related potential study. Cogn Emot [Internet]. 2017 Jan 2;31(1):127–138. [cited 2022 Jul 2] Available from: https://pubmed.ncbi.nlm.nih.gov/26403599/26403599 10.1080/02699931.2015.1089842

[5] Mealey L. Enhanced processing of threatening stimuli: the case of face recognition. Behav Brain Sci [Internet]. 1995;18(2):304–305. [cited 2022 Jul 2]. Available from: https://www.cambridge.org/core/journals/behavioral-and-brain-sciences/article/abs/enhanced-processing-of-threatening-stimuli-the-case-of-face-recognition/FF18D2107FA091B7A5824B5CFC98AF8D

[6] Verhoef MJ, Rose MS, White M, et al. Declining conventional cancer treatment and using complementary and alternative medicine: a problem or a challenge? Current Oncol [Internet]. 2008 Aug 25;15(Suppl 2):s101. Available from: /pmc/articles/PMC2528553/10.3747/co.v15i0.281 PMC2528553 18769571

[7] Chand NK, Subramanya HB, Rao GV. Management of patients who refuse blood transfusion. Indian J Anaesth Internet]. 2014 Sep 1 cited 2022 Jul 2;58(5):658. Available from. /pmc/articles/PMC4260316/25535432 10.4103/0019-5049.144680 PMC4260316

[8] Wu YC, Chen CS, Chan YJ. The outbreak of COVID-19: an overview. J Chin Med Assoc Internet]. 2020 cited 2022 Jul 4;83(3):217–220. Available from. https://journals.lww.com/jcma/Fulltext/2020/03000/The_outbreak_of_COVID_19__An_overview.3.aspx 32134861 10.1097/JCMA.0000000000000270 PMC7153464

[9] Kim JK, Crimmins EM, Sykes BL. How does age affect personal and social reactions to COVID-19: results from the National Understanding America Study. PLoS One Internet]. 2020 Nov 1 cited 2022 Jun 20;15(11):e0241950. Available from. https://journals.plos.org/plosone/article?id=10.1371/journal.pone.0241950 33170903 PMC7654776

[10] Caminati M, Vaia R, Furci F, et al. Uncontrolled asthma: unmet needs in the management of patients. J Asthma Allergy Internet]. 2021 cited 2022 Jul 19;14:457. Available from. /pmc/articles/PMC8104981/33976555 10.2147/JAA.S260604 PMC8104981

[11] Voineskos D, Daskalakis ZJ, Blumberger DM. Management of treatment-resistant depression: challenges and strategies. Neuropsychiatr Dis Treat Internet]. 2020 cited 2022 Jul 19;16:221 Available from. pmc/articles/PMC6982454 32021216 10.2147/NDT.S198774 PMC6982454

[12] Zervas E, Samitas K, Papaioannou AI, et al. An algorithmic approach for the treatment of severe uncontrolled asthma. ERJ Open Res Internet]. 2018 Jan 1 cited 2022 Jul 19;4(1):00125–2017. Available from. /pmc/articles/PMC5838355/29531957 10.1183/23120541.00125-2017 PMC5838355

[13] Xie Y, Baker J, Young T, et al. Therapy escalation following an elevated HbA1c in adults aged 45 years and older living with diabetes in Australia: a real-world observational analysis. Diabetes Care Internet]. 2020 Nov 1 cited 2022 Jul 16;43(11):e185–7. Available from. https://diabetesjournals.org/care/article/43/11/e185/35801/Therapy-Escalation-Following-an-Elevated-HbA1c-in 32928956 10.2337/dc20-0269

[14] Hurst JR, Dilleen M, Morris K, et al. Factors influencing treatment escalation from long-acting muscarinic antagonist monotherapy to triple therapy in patients with COPD: a retrospective THIN-database analysis. Int J Chron Obstruct Pulmon Dis Internet]. 2018 Mar 5 cited 2022 Jul 16;13:781. Available from.;:. [].: pmc/articles/PMC5842770/29551894 10.2147/COPD.S153655 PMC5842770

[15] Borges A. Ukraine war: children hiding in basement say they just don’t want to be bombed [Internet]. Euronews. 2022 cited 2022 Jul 22]. Available from 2022 Jul 22: https://www.euronews.com/2022/05/23/ukraine-war-children-hiding-in-basement-say-they-just-don-t-want-to-be-bombed

[16] Epstein J, Mitchell Simone T. Crowds of Ukrainians Taking Shelter in Subway [Internet]. Business Insider. 2022. [cited 2022 Jul 22]. Available from 2022 Jul 22]. https://www.businessinsider.com/video-ukraine-crowds-taking-shelter-in-subway-metro-station-2022-2

[17] Petherick A, Goldszmidt R, Andrade EB, et al. A worldwide assessment of changes in adherence to COVID-19 protective behaviours and hypothesized pandemic fatigue. Nature Human Behaviour [Internet]. 2021 Aug 3 cited 2022 Jul 12;5(9):1145–1160. Available from. https://www.nature.com/articles/s41562-021-01181-x 10.1038/s41562-021-01181-x 34345009

[18] Delussu F, Tizzoniid M, Gauvinid L. Evidence of pandemic fatigue associated with stricter tiered COVID-19 restrictions. PLOS Digital Health [Internet]. 2022 May 26 cited 2022 Jul 15;1(5):e0000035. Available from. https://journals.plos.org/digitalhealth/article?id=10.1371/journal.pdig.0000035 PMC9931343 36812519

[19] Haktanir A, Can N, Seki T, et al. Do we experience pandemic fatigue? Current state, predictors, and prevention. Curr Psychol Internet]. 2021 Oct 20 cited 2022 Oct 20;1:1–12. Available from. https://link.springer.com/article/10.1007/s12144-021-02397-w PMC8527300 34690475

[20] Meacci L, Primicerio M. Pandemic fatigue impact on COVID-19 spread: a mathematical modelling answer to the Italian scenario. Results Phys. 2021 Dec 1;31:104895. 34722137 10.1016/j.rinp.2021.104895 PMC8539631

[21] Fernandez-Lazaro CI, García-González JM, Adams DP, et al. Adherence to treatment and related factors among patients with chronic conditions in primary care: a cross-sectional study. BMC Fam Pract Internet]. 2019 Sep 14 cited 2022 Jul 15;20(1):1–12. Available from. https://bmcprimcare.biomedcentral.com/articles/10.1186/s12875-019-1019-3 31521114 PMC6744672

[22] Monmouth University. National: time to accept covid and move on? [Internet]. Monmouth University. 2022 cited 2022 Jul 12]. Available from 2022 Jul 12: https://www.monmouth.edu/polling-institute/documents/monmouthpoll_us_013122.pdf/

[23] Robinson-Lane SG. Adapting to chronic pain: a focused ethnography of black older adults. Geriatr Nurs (Minneap). 2020 Jul 1;41(4):468–473. 10.1016/j.gerinurse.2019.08.001 PMC8896290 31481258

[24] Sturgeon JA, Zautra AJ. Resilience: a new paradigm for adaptation to chronic pain. Curr Pain Headache Rep Internet]. 2010 Apr cited 2022 Jun 23;14(2):105. Available from. /pmc/articles/PMC4899321/20425199 10.1007/s11916-010-0095-9 PMC4899321

[25] Büssing A, Ostermann T, Neugebauer EA, et al. Adaptive coping strategies in patients with chronic pain conditions and their interpretation of disease. BMC Public Health Internet]. 2010 Aug 20 cited 2022 Jun 23;10(1):1–10. Available from. https://bmcpublichealth.biomedcentral.com/articles/10.1186/1471-2458-10-507 20727191 PMC2936426

[26] Seiler A, Jenewein J. Resilience in cancer patients. Front Psychiatry Internet]. 2019 Apr 1 cited 2022 Jul 12;10:208. Available from. /pmc/articles/PMC6460045/31024362 10.3389/fpsyt.2019.00208 PMC6460045

[27] Karoly P, Ruehlman LS. Psychological “resilience” and its correlates in chronic pain: findings from a national community sample. Pain Internet]. 2006 Jul cited 2022 Jun 11;123(1–2):90–97. Available from. https://journals.lww.com/pain/Fulltext/2006/07000/Psychological__resilience__and_its_correlates_in.13.aspx 16563626 10.1016/j.pain.2006.02.014

[28] Dezutter J, Casalin S, Wachholtz A, et al. Meaning in life: an important factor for the psychological well-being of chronically ill patients? Rehabil Psychol Internet]. 2013 Nov cited 2022 Jun 25;58(4):334–341. Available from. https://pubmed.ncbi.nlm.nih.gov/24295525/24295525 10.1037/a0034393 PMC4113206

[29] Sherman AC, Simonton S. Effects of personal meaning among patients in primary and specialized care: associations with psychosocial and physical outcomes. Psychol Health Internet]. 2012 Apr cited 2022 Jun 25;27(4):475–490. Available from. https://pubmed.ncbi.nlm.nih.gov/21722041/21722041 10.1080/08870446.2011.592983

[30] Jim HS, Richardson SA, Golden-Kreutz DM, et al. Strategies used in coping with a cancer diagnosis predict meaning in life for survivors. Health Psychol Internet]. 2006 Nov cited 2022 Jul 15;25(6):753. Available from. /pmc/articles/PMC2151209/17100503 10.1037/0278-6133.25.6.753 PMC2151209

[31] Szcześniak M, Świątek AH, Cieślak M, et al. Disease acceptance and eudemonic well-being among adults with physical disabilities: the mediator effect of meaning in life. Front Psychol. 2020 Oct 22;11:2432. 10.3389/fpsyg.2020.525560 PMC7643024 33192766

[32] Chabowski M, Junke M, Juzwiszyn J, et al. Adaptation to illness in relation to pain perceived by patients after surgery. J Pain Res Internet]. 2017 Jun 23 cited 2022 Jun 25;10:1447–1452. Available from. https://www.dovepress.com/adaptation-to-illness-in-relation-to-pain-perceived-by-patients-after–peer-reviewed-fulltext-article-JPR 28721086 10.2147/JPR.S129936 PMC5499957

[33] Benner JS, Glynn RJ, Mogun H, et al. Long-term persistence in use of statin therapy in elderly patients. JAMA Internet]. 2002 Jul 24 cited 2022 Jul 15;288(4):455–461. Available from.;():. [].: https://pubmed.ncbi.nlm.nih.gov/12132975/12132975 10.1001/jama.288.4.455

[34] Jackevicius CA, Mamdani M, v TJ. Adherence with statin therapy in elderly patients with and without acute coronary syndromes. JAMA Internet]. 2002 Jul 24 cited 2022 Jul 15;288(4):462–467. Available from. https://pubmed.ncbi.nlm.nih.gov/12132976/12132976 10.1001/jama.288.4.462

[35] Glader EL, Sjölander M, Eriksson M, et al. Persistent use of secondary preventive drugs declines rapidly during the first 2 years after stroke. Stroke Internet]. 2010 cited 2022 Jul 15;41(2):397–401. Available from.;():. [].: https://pubmed.ncbi.nlm.nih.gov/20075360/20075360 10.1161/STROKEAHA.109.566950

[36] Greer JA, Amoyal N, Nisotel L, et al. A systematic review of adherence to oral antineoplastic therapies. Oncologist Internet]. 2016 Mar 1 cited 2022 Jul 15;21(3):354–376. Available from. /pmc/articles/PMC4786357/26921292 10.1634/theoncologist.2015-0405 PMC4786357

[37] Galasso V, Pons V, Profeta P, et al. Gender differences in COVID-19 attitudes and behavior: panel evidence from eight countries. Proc Natl Acad Sci U S A. 2020 Nov 3;117(44):27285–27291. 33060298 10.1073/pnas.2012520117 PMC7959517

[38] Bodas M, Peleg K. Pandemic fatigue: the effects of the COVID-19 crisis on public trust and compliance with regulations in Israel. Health affairs (Project Hope). 2021 Aug 1;40(8):1225–1233. 34339236 10.1377/hlthaff.2021.00171

[39] Neuman S, Kennedy M. Firefighters gain ground on California wildfires : the two-way [internet]. NPR. 2017 cited 2022 Jul 12]. Available from 2022 Jul 12: https://www.npr.org/sections/thetwo-way/2017/10/16/557986665/firefighters-gain-ground-on-california-wildfires

[40] Fuller T, Pérez-Peña R. California wildfires death toll rises to 29 as vast region is scorched [Internet]. The New York Times. 2017 cited 2022 Jul 12]. Available from 2022 Jul 12: https://www.nytimes.com/2017/10/11/us/california-wildfires-firefighters.html

[41] Pagès-Puigdemont N, Mangues MA, Masip M, et al. Patients’ perspective of medication adherence in chronic conditions: a qualitative study. Adv Ther Internet]. 2016 Oct 1 cited 2022 Jul 16;33(10):1740–1754. Available from https://link.springer.com/article/10.1007/s12325-016-0394-6 27503082 PMC5055556

[42] Gonzalez JS, Peyrot M, McCarl LA, et al. Depression and diabetes treatment nonadherence: a meta-analysis. Diabetes Care. Internet]. 2008 Dec cited 2022 Jul 16;31(12):2398–2403. Available from. ;():.: https://pubmed.ncbi.nlm.nih.gov/19033420/19033420 10.2337/dc08-1341 PMC2584202

[43] DiMatteo MR, Lepper HS, Croghan TW. Depression is a risk factor for noncompliance with medical treatment: meta-analysis of the effects of anxiety and depression on patient adherence. Arch Intern Med Internet]. 2000 Jul 24 cited 2022 Jul 16;160(14):2101–2107. Available from.;():. [].: https://pubmed.ncbi.nlm.nih.gov/10904452/10904452 10.1001/archinte.160.14.2101

[44] Grenard JL, Munjas BA, Adams JL, et al. Depression and medication adherence in the treatment of chronic diseases in the United States: a meta-analysis. J Gen Intern Med. Internet]. 2011 Oct cited 2022 Jul 16;26(10):1175. Available from. /pmc/articles/PMC3181287/21533823 10.1007/s11606-011-1704-y PMC3181287

[45] Dobbins J, Campbell JH, Mann S, et al. Consequences of a precipitous U.S. withdrawal from Afghanistan perspective expert insights on a timely policy issue. 2018.

[46] Dalal S, Bruera E. End‐of‐life care matters: palliative cancer care results in better care and lower costs. Oncologist Internet]. 2017 Apr 1 cited 2022 Jul 16;22(4):361. Available from. /pmc/articles/PMC5388382/28314840 10.1634/theoncologist.2016-0277 PMC5388382

[47] Ahmedzai SH, Costa A, Blengini C, et al. A new international framework for palliative care. Eur J Cancer Internet]. 2004 Oct 1 cited 2022 Jul 16;40(15):2192–2200. Available from. http://www.ejcancer.com/article/S0959804904004976/fulltext 15454244 10.1016/j.ejca.2004.06.009

[48] Wiebe E, Shaw J, Green S, et al. Reasons for requesting medical assistance in dying. Can Family Physician Internet]. 2018 Sep 1 cited 2022 Jul 23;64(9):674. Available from. /pmc/articles/PMC6135145/PMC6135145 30209101

[49] Selby D, Bean S, Isenberg-Grzeda E, et al. Medical assistance in dying (MAiD): a descriptive study from a Canadian tertiary care hospital. Am J Hosp Palliat Care Internet]. 2020 Jan 1 cited 2022 Jul 23;37(1):58–64. Available from. https://pubmed.ncbi.nlm.nih.gov/31256607/31256607 10.1177/1049909119859844

[50] Brooks L. Health care provider experiences of and perspectives on medical assistance in dying: a scoping review of qualitative studies. Canad J Aging. 2019 Sep 1;38(3):384–396. 30626453 10.1017/S0714980818000600

[51] Katz MN. Lessons of the Soviet withdrawal from Afghanistan [Internet]. 2022 cited 2022 Jun 20]. Available from 2022 Jun 20: https://mepc.org/commentary/lessons-soviet-withdrawal-afghanistan

[52] Mendelson SE. Internal battles and external wars: politics, learning, and the Soviet withdrawal from Afghanistan. World Polit [Internet]. 1993 Apr cited 2022 Jun 20;45(3):327–360. Available from . ;():. [].: https://www.cambridge.org/core/journals/world-politics/article/abs/internal-battles-and-external-wars-politics-learning-and-the-soviet-withdrawal-from-afghanistan/9F5E65DF9347187A8A6F3321CAE8C3DC

[53] Hall S. Scholarly battles over the vietnam war. The Historical Journal [Internet]. 2009 cited 2022 Jun 17;52(3):813–829. Available from. https://www.cambridge.org/core/journals/historical-journal/article/abs/scholarly-battles-over-the-vietnam-war/1D3C0BBBB0F62FB59EC722F42A27BD26

[54] Israel A, El Jurdi K, Rubin DT. Treatment de-escalation in patients with inflammatory bowel disease. Gastroenterol Hepatol Internet]. 2019 cited 2022 Jul 16;15(6):335. Available from.;():. [].: /pmc/articles/PMC6676361/PMC6676361 31391803

[55] Pariente B, Laharie D. Review article: why, when and how to de-escalate therapy in inflammatory bowel diseases. Aliment Pharmacol Ther Internet]. 2014 Aug 1 cited 2022 Jul 22;40(4):338–353. Available from.;():. [].: https://onlinelibrary.wiley.com/doi/full/10.1111/apt.12838 24957164

[56] CDC. SARS-CoV-2 variant classifications and definitions [Internet]. CDC. 2022 cited 2022 Jun 23]. Available from 2022 Jun 23]. Available from 2022 Jun 23]. Available from 2022 Jun 23]. Available from 2022 Jun 23: https://www.cdc.gov/coronavirus/2019-ncov/variants/variant-classifications.html?CDC_AA_refVal=https%3A%2F%2Fwww.cdc.gov%2Fcoronavirus%2F2019-ncov%2Fvariants%2Fvariant-info.html

[57] Tebor C. Delta variant causes new lockdowns and coronavirus restrictions across the globe [Internet]. LA Times. 2021 cited 2022 Jun 20]. Available from 2022 Jun 20: https://www.latimes.com/world-nation/story/2021-07-01/delta-variant-worldwide-coronavirus-restrictions

[58] Beaubien J. Countries reimpose lockdowns with omicron spread [Internet]. NPR. 2022 cited 2022 Jun 20]. Available from 2022 Jun 20: https://www.npr.org/2022/01/08/1071542838/countries-reimpose-lockdowns-with-omicron-spread

[59] Meyer D. Lockdowns spread in Europe as Omicron COVID variant advances [Internet]. Fortune. 2021 cited 2022 Jun 20]. Available from 2022 Jun 20: https://fortune.com/2021/12/20/omicron-lockdown-netherlands-covid-europe-france-germany-italy-ireland/

[60] von Korff M, Tiemens B. Individualized stepped care of chronic illness. West J Emergency Med Internet]. 2000 cited 2022 Jun 28;172(2):133. Available from. /pmc/articles/PMC1070776/10.1136/ewjm.172.2.133 PMC1070776 10693379

[61] von Korff M, Glasgow RE, Sharpe M. ABC of psychological medicine: organising care for chronic illness. BMJ : British Medical Journal [Internet]. 2002 Jul 7 cited 2022 Jun 28;325(7355):92. Available from. /pmc/articles/PMC1123637/10.1136/bmj.325.7355.92 PMC1123637 12114242

[62] Simon S. The price of the surge [Internet]. Foreign Affairs. 2008 cited 2022 Jul 7]. Available from 2022 Jul 7: https://www.foreignaffairs.com/articles/iraq/2008-05-03/price-surge

[63] Chandrasekaran R. The Afghan surge is over [internet]. Foreign Policy. 2012 cited 2022 Jul 7]. Available from 2022 Jul 7: https://foreignpolicy.com/2012/09/25/the-afghan-surge-is-over/

[64] Radcliffe WS. The strategic surge in iraq: pretense or plan for success? 2007 Mar.

[65] Herren G. Flying man and falling man: remembering and forgetting 9/11. Faculty Scholarship [Internet]. 2014 cited 2022 Jun 20;3. Available from. http://www.exhibit.xavier.edu/english_faculty/3

[66] King M. North Korean sanctions evasion techniques. 2021 cited 2022 Jun 16]; Available from 2022 Jun 16: www.rand.org/about/principles.

[67] Kim MH. North korea’s cyber capabilities and their implications for international security. Sustainability (Switzerland). 2022 Feb 1;14(3):1744.

[68] Gause KE. North Korea’s provocation and escalation calculus: dealing with the Kim Jong-un Regime. 2015 Aug.

[69] Murphy L. Top 5 countries for crypto crime 2022 [Internet]. Coincub. 2022 cited 2022 Jul 4]. Available from 2022 Jul 4: https://coincub.com/top-5-countries-for-crypto-crime-2022/

[70] Pergolizzi J, Raffa RB, Rosenblatt MH. Opioid withdrawal symptoms, a consequence of chronic opioid use and opioid use disorder: current understanding and approaches to management. J Clin Pharm Ther Internet]. 2020 Oct 1 cited 2022 Jun 19;45(5):892–903. Available from.;():. [].: https://onlinelibrary.wiley.com/doi/full/10.1111/jcpt.13114 31986228

[71] Tobin DG, Holt SR, Doolittle BR. Responding to unsafe opioid use: abandon the drug, not the patient. J Gen Intern Med 2020 Internet]. 2020 Oct 9 cited 2022 Jul 8;36(3):790–791. Available from. https://link.springer.com/article/10.1007/s11606-020-06281-4 33037590 PMC7947064

[72] Wallace MS, Papp A Opioid withdrawal. Challenging cases and complication management in pain medicine [Internet]. 2022 Mar 7 cited 2022 Jun 23;15–20. Available from: https://www.ncbi.nlm.nih.gov/books/NBK526012/

[73] Tobore TO. Towards a comprehensive theory of non-cancer acute and chronic pain management: the critical role of reactive oxygen and nitrogen species in pain, and opioid dependence, addiction, hyperalgesia, and tolerance. Advances in Redox Research. [2021 Jul 1];2:100003.

[74] Hayhurst KP, Pierce M, Hickman M, et al. Pathways through opiate use and offending: a systematic review. Int J Drug Policy Internet]. 2017 Jan 1 cited 2022 Jun 16;39:1. Available from. /pmc/articles/PMC5234472/27770693 10.1016/j.drugpo.2016.08.015 PMC5234472

[75] Pierce M, Hayhurst K, Bird SM, et al. Insights into the link between drug use and criminality: lifetime offending of criminally-active opiate users. Drug Alcohol Depend Internet]. 2017 Oct 10 cited 2022 Jun 16;179:309. Available from. /pmc/articles/PMC5608072/28837946 10.1016/j.drugalcdep.2017.07.024 PMC5608072

[76] Siemaszko C. Women addicted to opioids turn to sex work in West Virginia [Internet]. NBC News. 2018 cited 2022 Jun 16]. Available from 2022 Jun 16: https://www.nbcnews.com/news/us-news/women-addicted-opioids-turn-sex-work-west-virginia-n868591

[77] Mitchell SG, Kelly SM, Brown BS, et al. Incarceration and opioid withdrawal: the experiences of methadone patients and out-of-treatment heroin users. J Psychoactive Drugs Internet]. 2009 cited 2022 Jul 8;41(2):145. Available from. /pmc/articles/PMC2838492/19705676 10.1080/02791072.2009.10399907 PMC2838492

[78] Jan S, Kimman M, Peters SAE, et al. Financial catastrophe, treatment discontinuation and death associated with surgically operable cancer in South-East Asia: results from the ACTION Study. Surgery (United States). 2015 Jun 1;157(6):971–982. 10.1016/j.surg.2015.02.012 25934082

[79] Das K, Banerjee M, Mondal GP, et al. Evaluation of socio-economic factors causing discontinuation of epilepsy treatment resulting in seizure recurrence: a study in an urban epilepsy clinic in India. Seizure. 2007 Oct 1;16(7):601–607. 17576079 10.1016/j.seizure.2007.04.008

[80] Sundell KA, Waern M, Petzold M, et al. Socio-economic determinants of early discontinuation of anti-depressant treatment in young adults. Eur J Public Health Internet]. 2013 Jun 1 cited 2022 Jul 15;23(3):433–440. Available from.;():. [].: https://academic.oup.com/eurpub/article/23/3/433/536576 21953063 10.1093/eurpub/ckr137

[81] Zhao L, Cesta CE, Pazzagli L. The role of socioeconomic factors on discontinuation of insulin during pregnancy—methodological challenges from a Swedish register-based study. Journal of Public Health (Germany) [Internet]. 2022 Feb 1 cited 2022 Feb 1;30(2):487–494. Available from. https://link.springer.com/article/10.1007/s10389-020-01307-x

[82] Washington Post. Latest Russia-Ukraine war news: live updates [Internet]. The Washington Post. 2022 cited 2022 Jun 18]. Available from 2022 Jun 18: https://www.washingtonpost.com/world/2022/06/19/russia-ukraine-war-putin-news-live-updates/

[83] Kaplan A. Tulsa shooting suspect killed doctor he blamed for back pain—and ‘anyone who got in his way,’ police say. Forbes. 2022 cited 2022 Jun 17]. Available from 2022 Jun 17: https://www.forbes.com/sites/annakaplan/2022/06/02/tulsa-shooting-suspect-killed-doctor-he-blamed-for-back-pain-and-anyone-who-got-in-his-way-police-say/?sh=21a9c3b12c9d

[84] Noland M. The (non) impact of UN sanctions on North Korea [Internet]. 2009 Feb cited 2022 Jun 24]. Available from 2022 Jun 24: https://www.files.ethz.ch/isn/96137/2009_02_The_(Non)_Impact_of_UN.pdf

[85] Min WJ, Han S. Economic sanctions against North Korea: the pivotal role of US–China cooperation. International Area Studies Review, 2020 23(2):177–193. 10.1177/2233865920901896

[86] Miller J. Apartheid South Africa and the collapse of the Portuguese empire [internet]. CWIHP. 2016 cited 2022 Jun 17]. Available from 2022 Jun 17: https://www.wilsoncenter.org/publication/apartheid-south-africa-and-the-collapse-the-portuguese-empire

[87] Levy BS, Leaning J. Russia’s war in Ukraine — the devastation of health and human rights. New England Journal of Medicine. 2022 Jun 29;387(2):102–105. [cited 2022 Jul 7]; Available from 2022 Jul 7]. Internet: https://www.nejm.org/doi/full/10.1056/NEJMp2207415 35767514

[88] DiPippo G. Strangling the bear? The sanctions on Russia after four months [Internet]. Center for Strategic and International Studies. 2022 cited 2022 Jul 8]. Available from 2022 Jul 8: https://www.csis.org/analysis/strangling-bear-sanctions-russia-after-four-months

[89] Raghupathi W, Raghupathi V. An empirical study of chronic diseases in the United States: a visual analytics approach to public health. Int J Environ Res Public Health Internet]. 2018 Mar 1 cited 2022 Mar 1;15(3):431. 10.3390/ijerph15030431 PMC5876976 29494555

[90] Park N, Park M, Peterson C. When is the search for meaning related to life satisfaction? Appl Psychol Health Well Being Internet]. 2010 Mar 1 cited 2022 Jun 28;2(1):1–13. Available from. https://onlinelibrary.wiley.com/doi/full/10.1111/j.1758-0854.2009.01024.x

[91] Yessick LR, Salomons T. The chronic disease helplessness survey: developing and validating a better measure of helplessness for chronic conditions. Pain Rep Internet]. 2022 Mar 14 cited 2022 Oct 21;7(2):E991. Available from: /pmc/articles/PMC8923572/35311028 10.1097/PR9.0000000000000991 PMC8923572

[92] Xie C, Li L, Li Y. Learned helplessness in renal dialysis patients: concept analysis with an evolutionary approach. Patient Prefer Adherence [Internet]. 2022 Aug 24;16:2301–2312. [cited 2022 Oct 21]. Available from: https://www.dovepress.com/learned-helplessness-in-renal-dialysis-patients-concept-analysis-with–peer-reviewed-fulltext-article-PPA 36042777 10.2147/PPA.S373134 PMC9420436

[93] oardar SD. Urban residential solid waste management in India: issues related to institutional arrangements. Public Works Management & Policy, 2000;4(4):319–30. 10.1177/1087724X0044006

